# P50, N100, and P200 Auditory Sensory Gating Deficits in Schizophrenia Patients

**DOI:** 10.3389/fpsyt.2020.00868

**Published:** 2020-08-27

**Authors:** Chen-Lan Shen, Tai-Li Chou, Wen-Sung Lai, Ming H. Hsieh, Chen-Chung Liu, Chih-Min Liu, Hai-Gwo Hwu

**Affiliations:** ^1^ Department of General Psychiatry, Tsao-Tun Psychiatric Center, Nanto, Taiwan; ^2^ Department of Psychology, College of Science, National Taiwan University, Taipei, Taiwan; ^3^ Department of Psychiatry, National Taiwan University Hospital and College of Medicine, Taipei, Taiwan; ^4^ Graduate Institute of Brain and Mind Sciences, National Taiwan University, Taipei, Taiwan

**Keywords:** event-related potentials, N100, P50, P200, paired click paradigm, schizophrenia, sensory gating

## Abstract

**Background:**

Sensory gating describes neurological processes of filtering out redundant or unnecessary stimuli during information processing, and sensory gating deficits may contribute to the symptoms of schizophrenia. Among the three components of auditory event-related potentials reflecting sensory gating, P50 implies pre-attentional filtering of sensory information and N100/P200 reflects attention triggering and allocation processes. Although diminished P50 gating has been extensively documented in patients with schizophrenia, previous studies on N100 were inconclusive, and P200 has been rarely examined. This study aimed to investigate whether patients with schizophrenia have P50, N100, and P200 gating deficits compared with control subjects.

**Methods:**

Control subjects and clinically stable schizophrenia patients were recruited. The mid-latency auditory evoked responses, comprising P50, N100, and P200, were measured using the auditory-paired click paradigm without manipulation of attention. Sensory gating parameters included S1 amplitude, S2 amplitude, amplitude difference (S1-S2), and gating ratio (S2/S1). We also evaluated schizophrenia patients with PANSS to be correlated with sensory gating indices.

**Results:**

One hundred four patients and 102 control subjects were examined. Compared to the control group, schizophrenia patients had significant sensory gating deficits in P50, N100, and P200, reflected by larger gating ratios and smaller amplitude differences. Further analysis revealed that the S2 amplitude of P50 was larger, while the S1 amplitude of N100/P200 was smaller, in schizophrenia patients than in the controls. We found no correlations between sensory gating indices and schizophrenia positive or negative symptom clusters. However, we found a negative correlation between the P200 S2 amplitude and Bell’s emotional discomfort factor/Wallwork’s depressed factor.

**Conclusion:**

Till date, this study has the largest sample size to analyze P50, N100, and P200 collectively by adopting the passive auditory paired-click paradigm without distractors. With covariates controlled for possible confounds, such as age, education, smoking amount and retained pairs, we found that schizophrenia patients had significant sensory gating deficits in P50-N100-P200. The schizophrenia patients had demonstrated a unique pattern of sensory gating deficits, including repetition suppression deficits in P50 and stimulus registration deficits in N100/200. These results suggest that sensory gating is a pervasive cognitive abnormality in schizophrenia patients that is not limited to the pre-attentive phase of information processing. Since P200 exhibited a large effect size and did not require additional time during recruitment, future studies of P50-N100-P200 collectively are highly recommended.

## Introduction

Schizophrenia is a brain disorder characterized by abnormal mental functions, including cognitive symptoms (1). Before the onset of cognitive and behavioral problems, a complex cascade of pathophysiological processes in the brains of schizophrenia patients had been noted, including alterations of gene expression, neurochemical-metabolic disturbances, alteration of brain connectivity, and impaired information processing (2). The combined changes ultimately lead to behavioral, cognitive, and emotional deficits, which are the clinical hallmarks of the disease.

Sensory gating describes neurophysiological processes of filtering out redundant or unnecessary stimuli during information processing, which potentially protects higher-order functions from being overloaded (3, 4). Sensory gating deficits have been proposed to cause sensory flooding and defective information processing to the brain and contribute to the symptoms of schizophrenia (5). To measure sensory gating deficits, mid-latency auditory evoked responses (MLAERs), comprised of P50, N100, and P200, have been studied while utilizing the auditory paired-click paradigm. The paired-click paradigm employs two identical auditory stimuli 500 ms apart to measure the amplitude changes in auditory evoked potentials between the two stimuli (S1 and S2), while the degree of sensory gating can be measured by its reduction with stimulus repetition, expressed either as the ratio between the P50 amplitude evoked by S2 divided by the amplitude evoked by S1 or as the absolute difference in amplitude between S1 and S2. An increased gating ratio (S2/S1) or decreased amplitude difference (S1-S2) are interpreted as auditory sensory gating deficit (6–9). The sensory gating deficit may be due to either one of the following two mechanisms: First, the S1 amplitudes are smaller in patients than in the control group. Second, the S2 amplitudes are attenuated less in patients (10).

Among the three MLAERs induced by the paired-click paradigm, P50, N100, and P200, have been studied in patients with different psychiatric disorders. P50 sensory gating deficit has been the most extensively documented in patients with schizophrenia (11–15). This deficit was also found in their first-degree relatives and individuals with ultra-high risk for schizophrenia and does not alter with clinical manifestations, so it has been regarded as an endophenotype for schizophrenia (5, 16). Furthermore, the P50 sensory deficit was also found in other mental illnesses, including Alzheimer’s disease, anti-social personality disorder, bipolar disorder, cocaine use disorder, panic disorder, posttraumatic stress disorder, and so forth (17–24).

In comparison to P50, which has been extensively documented in patients with schizophrenia, previous studies on N100 and P200 were relatively inconclusive. There are only few literatures of P200 with limited sample size in patients with schizophrenia (9, 25). Turetsky et al., using a large sample, measured N100 in 142 schizophrenia probands, 373 unaffected first-degree relatives, and 221 community comparison subjects, and proposed that there were no group differences for either S2 amplitude or the gating ratio (26). In addition, Rosburg, in a systemic meta-analysis of 29 auditory N100 gating studies in patients with schizophrenia, suggested a similar conclusion of decrease in the S1 amplitudes without significant change in the S2 amplitudes (27). In fact, the amplitude difference (S1–S2) was not noticed in above mentioned studies. Although Rosburg pointed out that ‘‘the gating ratio and alternatively used S1–S2 difference are less reliable measures than the individual amplitude measures” (page 2109), there were various N100 studies showing that patients with schizophrenia displayed decreased amplitude difference (S1–S2) instead of gating ratio (9, 28–31). Therefore, all gating measures, including amplitude difference, should be reported in future sensory gating studies, with higher ratios or smaller difference scores reflecting weaker gating.

Moreover, there are several methodological issues in the study of P50-N100-P200 auditory sensory gating deficits. The first is the issue of attention. While P50 reflects the pre-attentive filtering of information processing, N100 sensory gating may be related to the filtering mechanism involved in triggering of attention, and P200 gating may be related to the filtering mechanism involved in the allocation of attention (8, 9, 32). Accordingly, some N100/P200 studies used auditory stimulation distractors (20, 33), while some others used visual attention tasks (34–36). However, there are still some studies without distractors providing positive results(7, 24, 26, 32).

A second point is the acquisition of P50-N100-P200 *via* one paired-click paradigm collectively and clarifying their interrelationship in schizophrenia patients. Instead of schizophrenia, P50-N100-P200 has been studied in other psychiatric disorders, such as panic disorder (20), bipolar I disorder (21), antisocial personality disorder (24), cocaine users (22, 23), and autism spectrum disorders (32) in recent years.

The third point is the issue of filter settings. It should be noted that the filter settings of 29 studies reported in Rosburg’s systemic meta-analysis were diverse. For example, the two N100 studies with large numbers of subjects utilized different filter settings (1–50 Hz vs. 0.5–20 Hz) (26, 37), which made the comparison inappropriate. Methodological issues regarding different filter parameters have been mentioned (13, 31, 38, 39), and it will be appropriate to use similar filter settings that are generally accepted.

Besides methodological concerns, the relationship between these neurophysiological indexes and schizophrenic symptoms is also of concern. The correlation between clinical symptoms and P50-N100-P200 indexes is inconclusive in previous studies. For example, Adler et al. found that auditory sensory processing defects (P50/N100) in schizophrenia appear to be independent of negative symptoms measured by the SANS (40, 41). In contrast, some scholars have claimed that more severe negative symptoms are associated with more severe sensory gating in schizophrenia (42–44). In fact, P50 and N100 are often labeled as candidate endophenotypes or “trait” deficits in schizophrenia that are state-independent and enduring across different symptom statuses (13, 26, 45).

In the present study, with a relatively large sample of participants, we aimed to investigate whether patients with schizophrenia have P50, N100, and P200 gating deficits measured collectively by the auditory paired-click paradigm without control of attention. S1 amplitudes, S2 amplitudes, gating ratio (S2/S1), and amplitude difference (S1-S2) were explored. Correlations between the above parameters and PANSS were also evaluated. We hypothesized that patients with schizophrenia would display gating deficits of P50, N100, and P200.

## Methods

### Participants

The Institutional Review Board of the National Taiwan University Hospital approved this study. All participants gave written informed consent in accordance with the Declaration of Helsinki after the objective and procedures of the study were fully explained. We recruited patients with schizophrenia from the outpatient clinics of the Department of Psychiatry, National Taiwan University Hospital. Board certified psychiatrists made the diagnosis of schizophrenia based on the diagnostic criteria of the Diagnostic Statistical Manual Fourth Edition (DSM-IV). Patients with a diagnosis other than schizophrenia, such as bipolar affective disorders, organic mental disorders, and substance-related disorders, were excluded. The patients’ clinical symptomatology was evaluated using the Mandarin version of the Positive and Negative Syndrome Scale (PANSS) for schizophrenia (46). Patients met predetermined criteria for clinical stability, as they had been treated with the same antipsychotic medications for at least 3 months and had no inpatient stay during the past year. Medications were not experimentally controlled in this study. The healthy controls were recruited through advertisement with the requirement of neither having current or lifetime psychiatric diagnosis, nor had family history of psychotic disorders. Those with prior epileptic disorders, history of central nervous diseases, or traumatic brain injury were excluded from recruitment. Subjects were also required to refrain from smoking for at least 1 h prior to testing.

### Recording Environment

Electroencephalography (EEG) signals were recorded with a Quik-Cap (Compumedics Neuroscan, El Paso, TX, USA) from 32 scalp locations. All electrodes were placed according to the International 10–20 electrode placement standard, while electrodes placed at the tip of the nose (at Fpz) served as the reference and ground, respectively. Four additional electrodes were located above, below the left eye, and at the outer canthi of both eyes to monitor blinks and eye movements. Data were recorded on a Neuroscan ACQUIRE system (Compumedics Neuroscan, El Paso, TX, USA). Stimuli were digitized at a rate of 1 kHz and an on-line band-pass filter at 0.5–100 Hz, without applying 60-Hz notch filters. All electrode impedances were kept below 5 kΩ before recording.

### Testing Procedures

The auditory stimuli were generated by a Neuroscan STIM system, while auditory stimuli were presented to the subjects binaurally *via* foam insert earphones. The standard procedures for the auditory P50-N100-P200 paradigm were based on established protocols (6, 9, 11, 21, 30, 47). Before ERP recording, audiometry testing was used to exclude subjects who could not detect 40-dB sound pressure level tones at 500, 1,000, and 6,000 Hz presented binaurally. The participants had not smoked for at least 1 h before sessions and were instructed to lie down supinely in a comfortable recliner in a sound-attenuating, electrically shielded booth, and asked to relax with their eyes open and to focus on a fixation point. No tasks were performed during the test. EEG and stimuli were recorded continuously during the testing, and subjects were closely observed through a video monitor. If signs of sleep were detected visually or by slow way activity on EEG, the experimenter would talk briefly to the subject.

Online averaging was used to monitor the number of trials free from gross artifacts (defined as activities exceeding ± 100 μV in the −100–500 ms time-window following stimuli). Paired auditory clicks (1 ms, 85 dB) were presented every 8–12 s throughout the whole test session (average: 10 s), with a 500-ms interstimulus interval (39). When a minimum of 120 artifact-free trials had been obtained, the paired-click session was terminated, which took about 20–30 min.

### Offline Data Processing

Using Neuroscan Edit 4.5 software (Compumedics Neuroscan, El Paso, TX, USA), we followed the protocol regarding offline signal analysis formulated in previous publications (12, 30, 48). All data were processed by researchers who were blinded to the subject’s group assignment (49). Semiautomated procedures using the Tool Command batch processing Language (TCL) began with EOG artifact reduction through a built-in pattern-recognition algorithm (50). The data were epoched for the time window from -100 to 923 ms of the first click, with both S1 and S2 covered in the same epoch. All epochs containing activities surpassing ±50 μV were excluded, and retained pairs were compared between groups. To prevent temporal aliasing, we averaged the epochs digitally and band-pass-filtered them (10–50 Hz for P50, 1–50 Hz for N100 and P200) in the frequency domain. Peaks and preceding troughs were then detected at the Cz electrode using preset intervals automatically. The P50 peak was deﬁned as the largest positive deﬂection identified in the 40 and 75 ms poststimulus interval, with its amplitude defined as the difference between this peak and the preceding trough (not earlier than 30 ms poststimulus). The N100 peak was identiﬁed as the most negative deﬂection in the 80 to 150 ms poststimulus interval, and N100 amplitude was deﬁned as the absolute difference between the N100 peak and the preceding positive trough. The P200 peak was defined as the most positive deﬂection in the 150 to 250 ms poststimulus interval, with its amplitude measured as the absolute difference between the P200 peak and the preceding trough. Data from subjects with an S1 amplitude <0.5 μV were removed from further analysis. The P50, N100, and P200 parameters included S1 amplitude, S2 amplitude, amplitude difference (S1–S2), and gating ratio (S2/S1). A maximum gating ratio of 2 was applied to prevent outliers from disproportionately distorting the group mean (30, 48, 49, 51).

### Statistical Analysis

Statistical analyses were performed using IBM SPSS v22. For demographic characteristics, smoking amount, illness duration, CPZ equivalent dose, and ERP parameters, the results are presented as means and standard deviations (± SD). Chi-square tests were used for categorical variables when appropriate. Distributions were tested for normality using the Kolmogorov–Smirnov test with a significance level set at p=0.01. Distributions differing significantly from normality were normalized with a logarithmic transformation before proceeding with data analysis. The group differences for P50-N100-P200 parameters were tested using GLM repeated measures ANCOVA with age, education, smoking amount, and retained pairs as covariates, controlling for differences in those variables. Cohen’s d for presenting the effect size (the standardized difference between the two means) was computed between the control and schizophrenia groups with small, medium, and large effect sizes as the absolute value of Cohen’s d 0.2 to 0.5, 0.5 to 0.8, and ≥0.8, respectively.

We then examined correlations of S1 amplitude, S2 amplitude, gating ratio, and amplitude difference between P50, N100, and P200 in both the groups. We also calculated correlations of these parameters with the PANSS data in the schizophrenia group, while three PANSS structures were used: the three subscales classification (positive, negative, and general psychopathology total scores), Bell’s five-factor model (positive, negative, cognitive, emotional discomfort, and hostility components) (52), and Wallwork’s five-factor model (positive, negative, disorganized/concrete, excited, and depressed components) (53).

## Results

A total of 104 patients with schizophrenia and 102 healthy controls were recruited. Demographic and clinical characteristics are shown in **Table 1**. The two groups differed significantly in the age, years of education, smoking amount and retained pairs, but not in the distribution of gender. The schizophrenia group was older (39.7 ± 10.2 years vs. 31.8 ± 11.5 years, p<0.001), less educated (13.4 ± 2.8 years vs. 15.5 ± 3.2 years, p<0.001) and reported a much higher amount of smoking than the control group (0.154 ± 0.39 PPD vs. 0.029 ± 0.147 PPD). In addition, retained pairs differed between schizophrenia and control groups (107.7 ± 22.7 vs. 116.1 ± 20.6, p=0.006). In the schizophrenia group, the duration of illness was 14.1 ± 9.9 years, and the CPZ equivalent dose was 332.5 ± 229.8 mg. Among them, one patient was not treated with any antipsychotic, 20 patients were prescribed 1st generation antipsychotics, 71 patients were given 2nd generation antipsychotics (including 17 patients with clozapine), and 12 received a combination of 1st and 2nd generation antipsychotics. In terms of clinical severity shown by PANSS scores, the schizophrenia patients exhibited 11.8 ± 4.2, 15.3 ± 6.0, 25.4 ± 8.2, in positive symptoms, negative symptoms, and general psychopathology subscales, respectively.

**Table 1:** Demographics of control and patient groups (SD in parentheses).

	Controls	Patients	Statistics
	N=102	N=104	
Age	31.8(11.5)	39.7(10.2)	t=4.005, p<0.001**
Male gender (%)	45(44.1%)	52(50%)	χ2 (1)=0.715, p=0.398
Education (years)	15.4(3.2)	13.4(2.8)	t=4.858, p<0.001**
Smoking (PPD)	0.029(0.147)	0.154(0.39)	t=3.033, p=0.003*
Retained pairs	116.1(20.6)	107.7(22.7)	t=2.79, p=0.006*
			
Patients			
Age of onset		23.8(8.1)	
Duration of illness		14.1(9.9)	
CPZ equivalence		332.5(229.8)	
PANSS(P1 to P7)		11.8(4.2)	
PANSS(N1 to N7)		15.3(6.0)	
PANSS(G1 to G16)		25.4(8.2)	

*p < 0.05; **p < 0.001.

**Figure 1:**
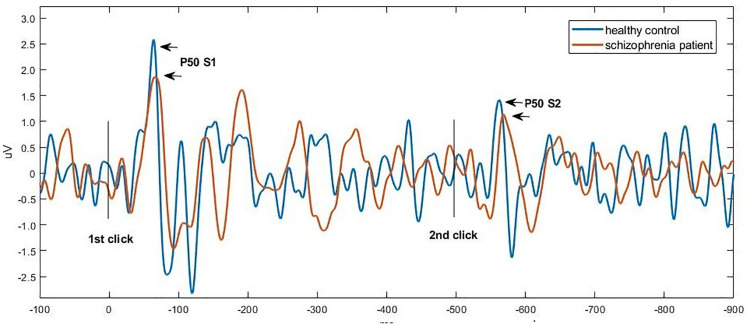
Grand average waveforms of P50 at Cz in a control subject (blue) vs. a schizophrenia participant (orange), respectively. Click stimuli were presented at time zero (first click) and at 500 ms (second click). The potential was filtered between 10 and 50 Hz to optimize scoring of the P50 component. The P50 peak was deﬁned as the largest positive deﬂection identified in the 40 and 75 ms poststimulus interval.

**Figure 2:**
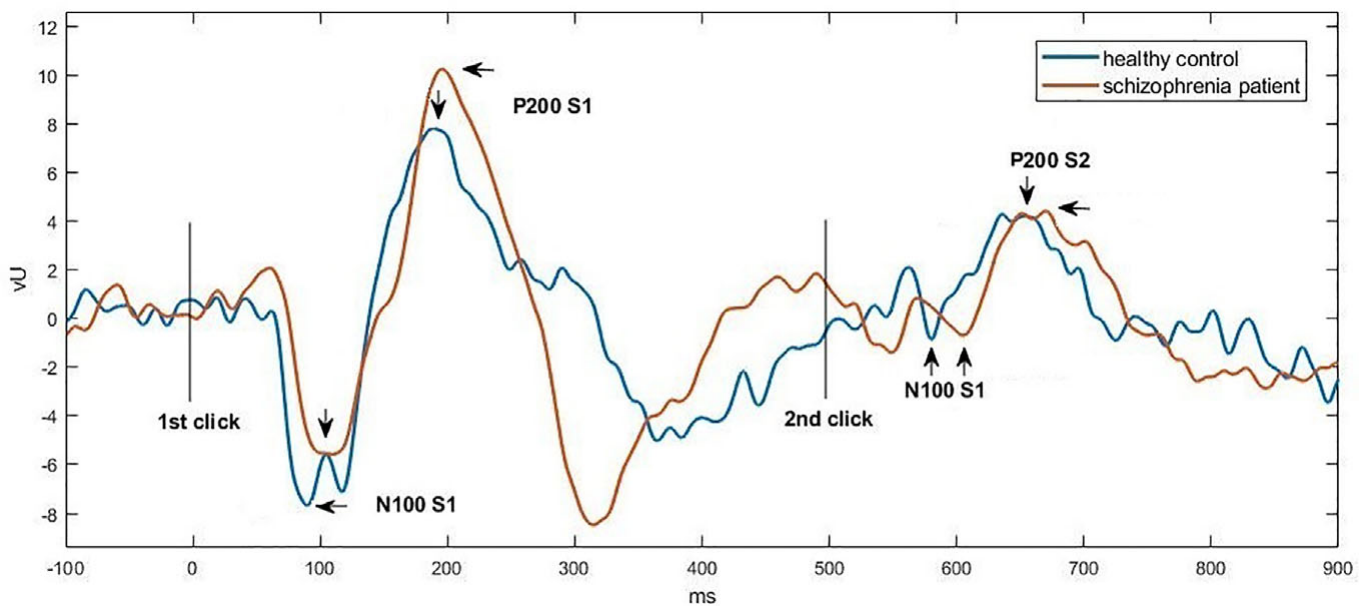
Grand average waveforms of N100-P200 at Cz in a control subject (blue) vs. a schizophrenia participant (orange), respectively. Click stimuli were presented at time zero (first click) and at 500 ms (second click). The potential was filtered between 1 and 50 Hz to optimize scoring of the N100 and P200 components. The N100 peak was identiﬁed as the most negative deﬂection in the 80 to 150 ms poststimulus interval, and the P200 peak was defined as the most positive deﬂection in the 150 to 250 ms poststimulus interval.

**Figures 1** and **2** show the grand average P50, N100, and P200 waveforms evoked by S1 and S2, respectively, in a control subject and a schizophrenia participant. Comparisons of event-related potentials are shown in **Table 2**. Distributions violating normality tests (all parameters other than P50 amplitude differences, N100 S2 amplitude, and P200 S2 amplitude) were normalized with a logarithmic transformation prior to data analysis. With age, education, smoking amount and retained pairs as covariates, the schizophrenia group had significantly larger gating ratios than the control group on P50 (p=0.019; Cohen’s d=0.358), N100 (p=0.002; Cohen’s d=0.453), and P200 (p=0.001; Cohen’s d=0.763). Besides, medium and large effect sizes were noted in the N100 amplitude difference (Cohen’s d=0.639) and P200 amplitude difference (Cohen’s d=0.841). Further analysis revealed different causes for these group differences in gating ratio. For P50, it was due to the elevated S2 amplitude and unchanged S1 amplitude; for N100 and P200, reversely, they were due to reduced S1 amplitude and unchanged S2 amplitude.

**Table 2:** Comparison of P50, N100 and P200 parameters between control and patient groups.

	Controls (n=102)		Patients (n=104)		GLM*		Cohen’s d
	Mean	SD	Mean	SD	F(4,200)	P	
P50 S1	2.328	1.006	2.316	1.136	0.006	0.94	0.011
P50 S2	1.042	0.628	1.362	0.874	9.275	0.003	0.42
P50 ratio (S2/S1)	0.518	0.381	0.664	0.432	5.652	0.019	0.358
P50 difference (S1-S2)	1.285	1.072	0.955	1.102	3.333	0.069	0.304
N100 S1	6.785	3.382	4.984	2.628	18.378	**<0.001**	0.595
N100 S2	2.171	1.190	2.370	1.573	1.039	0.309	0.142
N100 ratio	0.363	0.232	0.658	0.885	9.550	0.002	0.453
N100 difference	4.613	3.367	2.614	2.867	16.367	**<0.001**	0.639
P200 S1	11.8	6.051	7.904	4.036	29.529	**<0.001**	0.759
P200 S2	4.086	1.988	4.140	2.083	0.035	0.851	0.026
P200 ratio	0.390	0.215	0.597	0.318	26.318	0.001	0.763
P200 difference	7.714	5.371	3.674	3.93	27.693	**<0.001**	0.841

*Controlling for age, education, smoking amount, and retained pairs.

Regarding the relationship between P50, N100, and P200 parameters for the control and schizophrenia groups, the Pearson correlation coefficients are outlined in **Table 3**. In both the control group and the schizophrenia group, the P50 gating ratio did not correlate with the N100 or P200 gating ratio, but the N100 gating ratio was correlated significantly with the P200 gating ratio. For the difference scores and S1 amplitude, significant correlations between P50, N100, and P200 in both groups were noted. For S2, correlations between S2 amplitudes were only found in the schizophrenia group, while no significant correlations between P50 and N100 amplitude or between P50 and P200 amplitude in the control group were discovered.

**Table 3:** Pearson correlation coefficients (r) between P50, N100, and P200 ratios, between difference scores, and between amplitudes for the control and schizophrenia groups.

	Ratio		Difference	
	Control	Schizophrenia	Control	Schizophrenia
P50-N100	0.183	0.163	-0.529**	-0.473**
P50-P200	0.199	0.142	0.452**	0.322**
N100-P200	0.211*	0.396**	-0.780**	-0.706**
				
	**S1 amplitude**		**S2 amplitude**	
	**Control**	**Schizophrenia**	**Control**	**Schizophrenia**
P50-N100	-0.582**	-0.594**	-0.155	-0.457**
P50-P200	0.448**	0.352**	0.044	0.308*
N100-P200	-0.816**	-0.741**	-0.249*	-0.315*

*p < 0.05, 2-sided; **p < 0.001, 2-sided.

As for correlations between the P50-N100-P200 parameters and PANSS scores, we could not find any correlation of any individual PANSS items or three subscales with P50, N100, and P200 parameters (**Table 4**). Using Bell’s five-factor model (52), we found a negative correlation(r=−0.254, p=0.009) of P200 S2 amplitude with emotional discomfort factor (G2, G3, G6, G16). With Wallwork’s five-factor model (53), we found a negative correlation (r=−0.217, p=0.027) between the P200 S2 amplitude and the depressed factor (G2, G3, G6).

**Table 4:** Pearson correlation coefficients (r) between the P50/N100/P200 parameters and PANSS factors.

		P50 S1	P50 S2	P50 ratio	P50 difference	N100 S1	N100 S2	N100 ratio	N100 difference	P200 S1	P200 S2	P200 ratio	P200 difference
PANSS Subscales													
Positive	r p	0.043 0.666	0.044 0.66	0.058 0.56	0.01 0.923	-0.117 0.237	-0.006 0.949	-0.062 0.534	-0.104 0.294	0.023 0.813	-0.04 0.689	-0.04 0.684	0.045 0.649
Negative	r p	-0.099 0.319	-0.05 0.617	0.053 0.594	-0.062 0.529	0.032 0.749	-0.053 0.594	-0.021 0.829	0.058 0.557	-0.141 0.154	-0.007 0.945	0.165 0.095	-0.141 0.154
General	r p	-0.095 0.338	0.012 0.901	0.069 0.484	-0.108 0.277	0.004 0.965	0.039 0.693	-0.043 0.665	-0.017 0.86	-0.111 0.261	-0.191 0.052	-0.048 0.626	-0.013 0.896
Bell’s Five Factors													
Positive	r p	0.059 0.555	0.008 0.933	-0.017 0.865	0.054 0.589	-0.087 0.381	0.002 0.986	-0.034 0.734	-0.081 0.416	-0.001 0.99	-0.043 0.668	-0.015 0.883	0.021 0.83
Negative	r p	-0.193 0.05	-0.068 0.495	0.081 0.416	-0.145 0.141	0.115 0.246	-0.022 0.825	0.012 0.903	0.117 0.236	-0.187 0.057	-0.084 0.397	0.13 0.189	-0.148 0.135
Cognitive	r p	-0.018 0.853	0.037 0.706	0.058 0.557	-0.049 0.624	-0.077 0.436	-0.002 0.986	-0.132 0.181	-0.07 0.481	-0.052 0.599	-0.027 0.789	0.043 0.667	-0.04 0.69
Hostility	r p	-0.054 0.589	0.121 0.223	0.185 0.06	-0.151 0.126	-0.04 0.688	-0.035 0.726	-0.025 0.801	-0.017 0.86	-0.014 0.885	-.077 0.435	-0.079 0.426	0.026 0.792
Emotional discomfort	r p	–0.054 0.586	-0.02 0.84	0.019 0.848	-0.04 0.689	-0.005 0.963	0.079 0.428	-0.005 0.96	-0.047 0.633	-0.08 0.422	-0.254* 0.009	-0.085 0.391	0.053 0.592
Wallwork’s Five Factors													
Positive	r p	0.04 0.684	-0.027 0.785	-0.037 0.708	0.063 0.525	-0.024 0.808	-0.005 0.963	0.015 0.88	-0.02 0.844	-0.033 0.742	-0.029 0.771	-0.000 0.996	-0.018 0.854
Negative	r p	-0.185 0.054	-0.081 0.416	0.078 0.429	-0.131 0.184	0.111 0.261	-0.04 0.686	0.027 0.784	0.124 0.21	-0.185 0.06	-0.049 0.622	0.171 0.082	-0.164 0.097
Disorganized	r p	0.026 0.791	0.119 0.228	0.139 0.159	-0.067 0.496	-0.132 0.18	-0.036 0.718	-0.125 0.208	-0.102 0.304	-0.015 0.88	0.03 0.761	0.052 0.6	-0.031 0.751
Excited	r p	-0.054 0.589	0.121 0.223	0.185 0.06	-0.151 0.126	-0.04 0.688	-0.035 0.726	-0.025 0.801	-0.017 0.86	-0.014 0.885	-0.077 0.435	-0.079 0.426	0.026 0.792
Depressed	r p	-0.054 0.589	-0.008 0.935	0.024 0.812	-0.049 0.623	0.023 0.814	0.021 0.83	0.065 0.512	0.01 0.923	-0.08 0.419	-0.217* 0.027	-0.035 0.721	0.033 0.742

*p < 0.05.

## Discussion

To the best of our knowledge, this is the largest sample size to analyze P50, N100, and P200 collectively by adopting the passive auditory paired-click paradigm without distractors. Comparing 104 schizophrenia patients with 102 control subjects, we found that schizophrenia participants had significant P50 sensory gating deficits reflected by a larger P50 S2 amplitude and a larger P50 gating ratio. On N100, patients with schizophrenia demonstrated defective N100 sensory gating reflected by a smaller N100 S1 amplitude, larger N100 gating ratio, and smaller N100 amplitude difference. In addition, patients exhibited P200 sensory gating deficits reflected by smaller P200 S1 amplitude, a larger P200 gating ratio, and a smaller P200 amplitude difference. We found no correlations between sensory gating indices and schizophrenia positive or negative symptom clusters. However, we found a negative correlation (r=−0.217, p=0.027) between the P200 S2 amplitude and Bell’s emotional discomfort factor/Wallwork’s depressed factor.

Lijffijt et al. explored the effects of age, gender, education, and intelligence in 60 healthy subjects, and concluded that they might have influence on P50-N100-P200 (6). In addition, smoking has been found to normalize P50 sensory gating deficits transiently in schizophrenia patients (54–56). With rigorous covariates controlled for possible confounds, such as age, education, smoking amount and retained pairs, we found that schizophrenia patients had significant sensory gating deficits in P50-N100-P200, suggesting no effect of group difference of these possible confounders on current outcomes. Because subjects were required to refrain from smoking for at least 1 h prior to testing, the transient normalizing effect of cigarette smoking could be ignored.

In contrast to P50 and N100, which have been mentioned as essential biomarkers in schizophrenia, the P200 gating deficit in schizophrenia has been scarcely investigated (4, 15, 45, 57). One possible reason is the relevance of attention since many scholars have suggested that the distraction paradigm may enhance the detection of abnormal gating in patients with schizophrenia (34). Consequently, visual and auditory distractors were given in the N100/P200 trials (20, 33, 35, 36). However, Rosburg et al. suggest that the active control of attention is difficult in sensory gating experiments, since long intervals of no stimulation between the paired clicks are a necessary component of these experiments (58). In fact, there were only a few schizophrenia studies that analyzed auditory P50-N100-P200 using a paired-click paradigm without distractors (9, 30, 48). Boutros et al. examined P50-N100-P200 in 23 patients with schizophrenia and age/gender-matched healthy control subjects and concluded that patients with schizophrenia had demonstrable habituation or sensory gating difficulties throughout the mid-latency range of information processing, including N100/P200 (9). This study, using larger sample size, confirmed the viewpoint of the Boutros group. In addition, P200 had a larger effect size of gating ratio (Cohen’s d=0.763) and amplitude difference (Cohen’s d=0.841) than the gating ratio and amplitude difference of N100 and P50, indicating the presence of P200 gating deficits in schizophrenia patients. Light et al. explored a comprehensive study of neurophysiological and neurocognitive biomarkers for use as neural substrates and genomic studies in schizophrenia, including four parameters of P50 and N100, respectively (16), and concluded that they could be considered as endophenotypes. Previous studies have reported that P200 has better reliability than P50 and N100 (7). Since P200 exhibited a larger effect size and did not require additional time during recruitment, future studies of P50-N100-P200 are highly recommended.

A meta-analysis by de Wilde et al. revealed that the effect size for P50 sensory gating is large, with a measure across studies of Cohen’s d=1.28. However, the differences were heterogeneous and not the same across all the studies (13, 39). This study recruited a sample size of 104 schizophrenia patients and 102 control subjects, which exceed most of the P50 literature, confirmed the role of P50 sensory gating deficits in schizophrenia. Sensory gating deficits were mainly due to differences in S2 (Cohen’s d=0.42) rather than S1, which was confirmed by other studies (11, 59), despite the proposition of some researchers that S1 amplitudes largely determine differences between normal subjects and schizophrenia patients on P50/N100 sensory gating (60). For N100, we found medium effect sizes for S1 amplitude (Cohen’s d=0.595) and amplitude difference (Cohen’s d=0.639), with a small effect side on gating ratio (Cohen’s d=0.453). There were no group differences in S2. Our N100 results corroborate the findings of Turetsky et al. (26) and were in line with Rosburg’s meta-analysis study (27). Interestingly, our results were different from those of Light et al., who proposed that the P50/N100 amplitude difference and gating ratio have only a limited effect size compared to the S1 and S2 amplitudes of P50/N100. Differences in methodology might be a possible interpretation (39).

Previous studies have extensively documented elevated P50 gating ratio in schizophrenia patients, but it remains unclear whether this is due to smaller S1 amplitude or larger S2 amplitudes in patients than in the control group. As conceptualized by Boutros et al., “ERPs elicited in the paired stimuli procedure reflect the abilities of the nervous system to both (i) “gate in” novel, or salient, information (i.e. stimulus registration, as measured by ERP amplitude to S1) and (ii) filter out extraneous information (i.e. repetition suppression, as measured by ERP amplitude suppression at S2)” (61, 62). S1 and S2 responses may index separate psychological phenomenon, while S1 reflects information registration of the stimuli and S2 reflects information habituation to the repeating stimulus (34, 63). Our results revealed that sensory gating deficits were due to larger S2 amplitudes in patients than in the control group in P50 as well as smaller S1 amplitudes in patients than in the control group in N100 and P200, suggesting that P50 and N100/P200 gating deficits may be due to different neurophysiological mechanisms. This study is the first one to delineate a unique pattern of P50-N100-P200 sensory gating deficits in schizophrenia patients: i.e. repetition suppression deficits in P50 and stimulus registration deficits in N100/200.

Concerning the relationship between P50, N100, and P200 parameters in **Table 3**, the P50 gating ratio does not correlate significantly with those of the N100 and P200 (21, 64, 65) suggesting that they tap into the integrity of different underlying mechanisms. However, regarding the amplitude difference score and S1 amplitude, rather than the S2 amplitude, P50 correlated significantly with N100 and P200. These findings not only corroborate that processing of auditory information differs between S1 (information registration of the stimuli) and S2 (information habituation to the repeating stimulus), but also validate the viewpoint by Boutros that difference measure is more closely related to S1 amplitude (62). Because S1 amplitude and S2 amplitude of P50 are involved in the different cognitive domain deficits (66), distinguishing the source of change helps to clarify the underlying mechanisms of sensory gating deficits.

Regarding the underlying mechanism of sensory gating, the role of alpha-7 nicotinic system on P50 has been documented (45, 56, 67, 68), and the neuroanatomy of P50 involves the hippocampal, temporal, and frontal lobe regions (15, 62, 69–73). To model abnormalities in the P50, N100, and P200 in schizophrenia, Connoly et al. analyzed the effects of ketamine P20, N40, and P80 event-related potential components in mice. Ketamine increased the P20/N40 amplitude and decreased the P80 amplitude. Although the effects of ketamine in mice P80 were consistent with P200 ERP changes in schizophrenia, the effects of ketamine in mice P20/N40 are inconsistent with alterations in the corresponding P50 and N100 in schizophrenia. Therefore, NMDA dysfunction may contribute to P200 deficits, but not P50-N100, in schizophrenia (74). More efforts are needed to delineate the underlying mechanism of sensory gating besides P50 in order to develop novel schizophrenia therapeutics (75–77).

Regarding the correlations between sensory gating measures and schizophrenia symptom clusters, some scholars have claimed that more severe negative symptoms are associated with more severe sensory gating in schizophrenia (42–44). However, others failed to demonstrate a relationship between negative symptoms and P50 sensory gating (9, 29, 40, 78–80). Regarding positive symptoms and sensory gating, no previous studies have been noted (42, 78, 80). Our results revealed that, besides the negative correlation of P200 S2 amplitude with Bell’s emotional discomfort factor (G2, G3, G6, G16) and Wallwork’s depressed factor (G2, G3, G6), which needs to be further replicated, we could not find any correlation between any individual PANSS items or other PANSS factor dimensions with P50, N100, and P200 parameters. Interestingly, Boutros et al., in their P50-N100-P200 research, found that none of the P50 or N100 derived sensory gating measures correlated with any of the PANSS derived scales, but the P200 gating ratio measure correlated positively with the Bell’s emotional discomfort symptom cluster (9). Previous studies exploring neuroanatomy of symptom dimensions in schizophrenia focused on major symptom factors rather than Bell’s emotional discomfort factor or Wallwork’s depressed factor (81–83). Therefore, our finding should await validation prior to further speculation.

Other reasons for scanty relationships between ERPs and clinical symptoms of schizophrenia, according to Ford’s comment, may include the possibilities that ERP studies are not sensitive to schizophrenia symptoms or mechanisms, patients’ subjective experiences are difficult to report and fathom, antipsychotics dissociate the symptoms from the neurobiology or the symptoms are nonspecific to schizophrenia (84). To summarize, our findings indicate that P50-N100-P200 sensory gating may reflect a more stable trait than clinical symptoms that vary over time (21, 85). The absence of clinical correlation with sensory gating deficits might be considered as the characteristic of endophenotypes, while the criteria for a candidate endophenotype include state-independence, that is, it manifests whether or not the illness is active (16, 86).

Some limitations of the current study are worth noting. First, antipsychotic medications, often prescribed to improve positive symptoms, were not experimentally controlled in this cross-sectional study. Although some studies showed that second generation antipsychotics, especially clozapine, may normalize P50 gating ratio in schizophrenia patients (80, 87), after comparing the patients taking clozapine (n=17) and non-clozapine antipsychotics (n=87), we found no significant difference between these two subgroups in any gating ratio (P50 gating ratio: t=0.963, p=0.344; N100 gating ratio: t=−0.453, p=0.652; P200 gating ratio: t=0.674, p=0.356). Our findings are supported by the viewpoints that P50/N100 sensory gating deficits were not influenced by antipsychotics (14, 27, 44, 88, 89). Since P200 involves attention allocation processes and antipsychotics may have an impact on information processing speed, medication status may confound our findings. Therefore, longitudinal studies will be needed to clarify the effect of medication. Also, novel techniques besides time-domain grand average analysis have not been used, such as phase locking analysis, frequency domain analyses, and so on (28, 60, 90, 91). For example, gamma spectrum oscillations mostly contributed to the prediction of the P50, and theta spectrum oscillations mostly to the N100 (28, 60).

In conclusion, the present study is the largest one to acquire auditory P50-N100-P200 collectively in a task-free pair-click paradigm without distractors, and the first one to delineate a unique pattern of sensory gating deficits in schizophrenia patients: i.e. repetition suppression deficits in P50 and stimulus registration deficits in N100/200. Schizophrenia patients demonstrated significant sensory gating deficits in P50-N100-P200, even after controlled for possible covariates, such as age, education, smoking amount and retained pairs. These results suggest that sensory gating is a pervasive abnormality in schizophrenia patients that can be detected throughout the entire mid-latency range of information processing and is not limited to the pre-attentive stages. We corroborated the findings of very few previous studies on P200 gating deficits in schizophrenia patients. P50, N100, and P200 sensory gating deficits in schizophrenia patients may be associated with different mechanisms and warrants further investigation.

## Data Availability Statement

The datasets generated for this study are available on request to the corresponding author.

## Ethics Statement

The studies involving human participants were reviewed and approved by National Taiwan University Hospital (NTUH) Institute Review Board. The patients/participants provided their written informed consent to participate in this study.

## Author Contributions

MH reviewed the literature and designed this study. C-LS worked out all the ERP technical details and wrote the manuscript. W-SL and T-LC contributed to interpreting the results and worked on the manuscript. C-CL and C-ML helped to recruit subjects and were involved in the clinical and diagnostic assessments. H-GH provided funding and oversaw the clinical trial. All authors discussed the results and commented on the manuscript.

## Funding 

This work was supported by the Ministry of Science and Technology, Taiwan (Grant number MOST-108-2320-B-002-055; MOST-108-2314-B-002-010; PI: MH).

## Conflict of Interest

The authors declare that the research was conducted in the absence of any commercial or financial relationships that could be construed as a potential conflict of interest.
